# Residual monomer release from different acrylic-based dental materials: An *in vivo* evaluation

**DOI:** 10.6026/973206300214595

**Published:** 2025-12-15

**Authors:** Anil Kumar Venkeshwarlu James, Vivek Narra, Kalyana Ponangi, Sheeba Glory Chowdary, Mahesh Tagore T, Hardik Dholakia

**Affiliations:** 1 Department of Jefferson Dental & Orthodontics, 907 S WW White RD, San Antonio, Texas, USA; 2 General Dentist, Familia Dental, Springfield Illinois, USA; 3 General dentist, Noble Dental Community Clinics, Wautoma, Wisconsin, USA; 4 University of Texas San Antanio School of Dentistry, Texas, USA; 5 Department of Oral & Maxillofacial Pathology, SVS Institute of Dental Sciences, Mahabubnagar, Telangana, India; 6 Thundermist health centre, Woonsocket, USA

**Keywords:** Methylmethacrylate, acrylic resins, thermoformed sheets, leachability, HPLC

## Abstract

Primary concern from residual monomer and other leachable components from denture base resins is allergic and sensitivity reactions among patients. Therefore, it is of interest to evaluate the leaching ability of residual methylmethacrylate monomer from heat polymerising acrylic, auto polymerising acrylic, auto polymerising acrylic cured under pot pressure, and thermoformed sheets in 40 children aged 7-14 years. Saliva samples were collected at 5 minutes, 1 hour, 24 hours, and 1 month after appliance insertion and analyzed by High Performance Liquid Chromatography. Results revealed significantly higher monomer release from auto polymerising acrylic materials, followed by heat cure, while thermoformed sheets showed no detectable release. The findings indicate thermoformed sheets are safer and more clinically acceptable alternatives for pediatric appliances.

## Background:

Polymeric materials are widely used in dentistry for the fabrication of denture bases, orthodontic appliances, and temporary restorations. However, despite their versatility, polymers are associated with several limitations, including incomplete polymerization, degradation, and release of potentially harmful substances in the oral cavity [1]. One of the earliest concerns identified was the occurrence of allergic and sensitivity reactions among patients due to residual monomer and other leachable components from denture base resins. Clinical manifestations such as burning mouth syndrome have been linked to these leachables, highlighting the importance of biocompatibility in prosthetic materials [2]. The polymerization method significantly influences the amount of residual monomer present in acrylic resins. Studies have shown that auto-polymerized resins generally exhibit higher levels of unreacted monomer compared to heat-polymerized materials, thereby posing a greater risk of cytotoxicity and allergic response [3]. Apart from residual monomer, the leaching of plasticizers from soft lining materials has also been reported. These plasticizers are prone to biodegradation in the oral environment, which can further compromise both the biological safety and the longevity of the prosthesis [4]. Another compound of concern is dibenzoyl peroxide, a polymerization initiator, which may be released from polymethyl methacrylate denture base resins. *In vitro* evaluations have demonstrated detectable levels of this substance, raising additional concerns regarding its biocompatibility [5]. Furthermore, dental polymer networks undergo hygroscopic and hydrolytic degradation when exposed to the moist oral environment. This process alters their physical and mechanical properties while facilitating the release of residual substances into saliva [6]. The formulation of dental composites and acrylics plays a direct role in their degradation profile. Specific ingredients and polymer network structures influence the extent of hydrolyzed resin-derived products released during function, emphasizing the importance of material selection [7]. In addition, commercially available temporary restorative resins have also shown measurable levels of leachable monomers and plasticizers. These findings indicate that the issue of biocompatibility is not limited to permanent prosthetic materials but extends to temporary ones as well [8]. Therefore, it is of interest to assess the residual monomer release from different acrylic-based dental materials.

## Methodology:

This study was carried out on children aged between 7 and 14 years who reported to the Department of Pedodontics and Preventive Dentistry, Sri Venkata Sai Institute of Dental Sciences, Mahabubnagar. Only children in good general health without any dental pain or abscess were included. The selection criteria comprised children within the specified age group requiring interceptive orthodontic treatment, with an assurance that the appliance would be worn for at least one month. Cooperative children with motivated parents free from any physical or mental disabilities, and those who had already completed emergency, restorative, and endodontic treatments were considered eligible. A total of 40 children were included and divided equally into four groups: appliances fabricated with heat polymerising acrylic resin (Group 1), autopolymerising acrylic resin cured under pot pressure (Group 2), autopolymerising acrylic resin (Group 3), and thermoforming sheets containing methylmethacrylate (Group 4).After appliance delivery, saliva samples were collected at intervals of 5 minutes, 1 hour, 24 hours, and 1 month. Each child was seated upright with the head tilted forward, and 2 ml of saliva was collected by asking them to spit the pooled saliva every 30 seconds into sterile screw-capped containers. Samples were also collected before appliance insertion to serve as baseline controls. The appliances were fabricated using standard laboratory procedures according to manufacturer's instructions. Heat polymerised acrylic resin was prepared using a 35 g:14 ml powder-liquid ratio, packed at dough stage, processed with a hydraulic press, and polymerised at 70°C for 90 minutes followed by 100°C for 30 minutes. Autopolymerising resin was mixed in the recommended ratio of 10 g powder to 7 ml liquid, packed at dough stage, and allowed to harden at room temperature with a processing time of 10-14 minutes. For the pot-pressure group, the same resin mixture was processed under 2000 Psi pressure in water at 50°C for 15 minutes. Thermoforming sheets containing polymethyl methacrylate were processed under a vacuum thermoforming machine at 195°C for 120 seconds with a cooling time of 3 minutes. All salivary samples were analyzed for methylmethacrylate release using reverse-phase high-performance liquid chromatography (HPLC). Methyl acrylate (0.1 µg/ml, 50 µl in acetonitrile) was used as an internal standard, with the lower detection limit set at 0.05 µg/ml. A 20 µl aliquot of saliva was injected into a 4.6 mm x 25 cm LiChrosorb RP-18 packed column. Chromatographic separation was performed under isocratic elution using a mobile phase of 30% acetonitrile in 50 mmol/L phosphate buffers (pH 2.5) at a flow rate of 1.0 ml/min and maintained at 50°C. Ultraviolet absorption of the elite was recorded at 210 nm, and the concentration of methylmethacrylate was quantified based on calibration graphs prepared from standard solutions. The results were tabulated and subjected to statistical analysis using Tukey's multiple hoc test and paired t test to determine both intergroup and intragroup differences.

## Results:

The study included 40 kids, 26 of whom were boys and 14 of whom were girls, ages 7 to 14. Ten kids each made up each of the four equal groups into which the participants were split. According to the age distribution, the majority (n=16) were in the 11-12 year range, followed by the 7-8 year (n=11), 9-10 year (n=9), and 13-14 year (n=4) age groups. Table 1 (see PDF) displays the sample distribution for each group by age and gender. At five minutes, one hour, twenty-four hours, and one month, the leachability of methyl methacrylate (MMA) was evaluated. Appliances made with heat-polymerizing acrylic (Group 1) released 0.15 ± 0.14 µg/ml at 5 minutes and 0.43 ± 0.19 µg/ml at 1 hour, according to the results, while there was no discernible release at 24 hours or 1 month. Autopolymerizing acrylic (Group 2) showed higher levels of release, with 0.34 ± 0.13 µg/ml at 5 minutes and 1.16 ± 0.73 µg/ml at 1 hour. In contrast, autopolymerizing acrylic that was cured under pot pressure (Group 3) showed 0.44 ± 0.30 µg/ml at 5 minutes and 1.02 ± 0.52 µg/ml at 1 hour. The methyl methacrylate-containing thermoformed sheet (Group 4), on the other hand, showed no discernible release during any of the assessed time periods. Group 1 showed a mean difference of 0.28 ± 0.25 µg/ml when comparing differences between 5 minutes and 1 hour, Group 2 showed the largest increase with 0.82 ± 0.75 µg/ml, Group 3 recorded 0.58 ± 0.36 µg/ml, and Group 4 stayed at 0.00 µg/ml. Table 2 (see PDF) summarises the significant pairwise differences between the groups that were found by statistical analysis using Tukey's post hoc test. Overall, the findings show that appliances made with autopolymerising acrylic had the highest MMA release, followed by autopolymerizing acrylic cured under pot pressure and heat polymerising acrylic. Thermoformed sheets, on the other hand, did not exhibit any release at any time.

## Discussion:

The biodegradation of dental polymers is a critical issue that influences their long-term clinical performance. Enzymatic degradation has been demonstrated to play a significant role, with cholesterol esterase identified as a key factor in breaking down commercial dental composites [9]. Similarly, salivary esterase activity has been shown to accelerate the biodegradation process, further contributing to the release of breakdown products in the oral cavity [10]. Mechanical stresses in the oral environment also influence degradation. Resin composites are susceptible to fatigue, wear and failure over time, which can increase the rate of monomer and additive release [11]. This supports the broader understanding that biodegradation of acrylic-based resins is a multifactorial process involving both chemical and mechanical pathways [12]. One major concern is the release of residual monomers into saliva. Early investigations confirmed the presence of methyl methacrylate monomer in the oral environment of patients wearing acrylic appliances, suggesting continuous leaching during use [13]. Additional studies highlighted the leaching of formaldehyde and methyl methacrylate, both of which possess cytotoxic potential [14]. The manipulation and finishing of denture base resins have also been shown to influence residual monomer levels. In situ studies demonstrated that improper processing or polishing can significantly increase the amount of monomer released intraorally [15]. Similarly, laboratory evaluations confirmed that acrylic resins in contact with artificial saliva continue to release measurable amounts of monomers, further supporting clinical observations [16]. Apart from monomers, plasticizers incorporated into soft denture liners can also leach out. Both *in vivo* and *in vitro* experiments revealed considerable loss of plasticizers, which not only compromises material properties but also introduces potential toxicological r isks [17]. Longitudinal clinical studies further demonstrated that dentures in function can release organic additives, highlighting the persistent nature of leachability [18]. The cytotoxicity of eluates from denture base resins has been widely documented. Light-polymerized resins, for instance, have been shown to release components capable of causing adverse cellular responses [19]. The cytotoxic potential of residual methyl methacrylate was also demonstrated in comparative studies between heat-cured and autopolymerized resins, with the latter generally releasing higher amounts [20]. At the cellular level, acrylate and methacrylate monomers can induce cytotoxicity through mechanisms such as intracellular calcium mobilization, which is related to their hydrophobicity and interaction with cell membranes [21]. *In vitro* biocompatibility testing further confirmed that the polymerization cycle and post-polymerization treatments strongly influence the degree of cytotoxicity observed in cell cultures [22]. Analytical techniques such as HPLC have also been employed to quantify residual monomers, confirming their presence in clinically relevant amounts even after standard curing cycles [23]. With the advent of newer processing techniques, CAD/CAM resins have been proposed as alternatives. Recent evidence suggests that CAD/CAM-fabricated resins possess lower residual monomer content and better mechanical properties compared to conventionally heat-cured resins, offering a potential solution to some of the limitations of traditional materials [24, 25].

## Conclusion:

Thermoformed sheets proved to be the safest, most biocompatible, and patient-friendly option. This suggests that thermoplastic materials may represent a more reasonable alternative to conventional acrylics in pediatric appliance fabrication. However, long-term clinical studies with larger sample sizes are warranted to validate these findings and to establish definitive recommendations.
